# Postpartum Stressors Cause a Reduction in Mechanical Brush Use in Dairy Cows

**DOI:** 10.3390/ani11113031

**Published:** 2021-10-22

**Authors:** Benjamin Lecorps, Allison Welk, Daniel M. Weary, Marina A. G. von Keyserlingk

**Affiliations:** Animal Welfare Program, Faculty of Land and Food Systems, 2357 Main Mall, University of British Columbia, Vancouver, BC V6T 1Z6, Canada; benjamin.lecorps@gmail.com (B.L.); allison.welk@gmail.com (A.W.); danweary@mail.ubc.ca (D.M.W.)

**Keywords:** cow-calf separation, emotion, animal welfare, animal well-being

## Abstract

**Simple Summary:**

Dairy cows face many stressors in the weeks immediately before and after calving. Reduced expression of low-resilience behaviors, such as the use of a mechanical brush, can be used to make inferences about affective states. Our study aimed at assessing the effects of stressors associated with parturition by following mechanical brush use after calving and after separation from their calf. We found that cows reduced their use of the brush postpartum. We also found that separation from the calf induced a reduction in brush use, suggesting that it has negative effects on cows.

**Abstract:**

Dairy cows are often subjected to multiple post-partum stressors but how these stressors impact cows’ affective states remain poorly understood. Negative affective states are often associated with reduced expression of low-resilience behaviors, so we explored whether cows would reduce their use of a brush after calving. Before calving, cows were offered the opportunity to use a mechanical brush once a week for 10 min. In Experiment 1, we explored whether cows reduced their use of a mechanical brush after parturition (compared to prepartum values) when subjected to the myriad of stressors typically experienced by cows at this time. In Experiment 2, we assessed the effect of cow–calf separation. Results from Experiment 1 showed that cows displayed a reduced brush use following parturition compared to the week before calving. In Experiment 2, we showed that cows given more time to bond with their calf, and who were separated more recently from their calf, showed a more pronounced reduction in brush use. Cows provided part-time contact with their calf for 29 days also reduced their brush use when they were permanently separated from their calf on day 30 after calving. These results suggest that cows experienced anhedonia and point to new directions for research on dairy cow affective states.

## 1. Introduction

Parturition in dairy cows is associated with many stressors including pain [1], unstable social environments [2], and separation from the newborn calf [3], making it a particularly challenging time. All these stressors are likely to induce negative affective states. For instance, work in other species has shown that pain (e.g., mice: [4]), social stress (e.g., rats: [5]), and separation from the offspring (e.g., laboratory rodents: [6]) are all associated with persistent negative affective states (i.e., negative mood).

Stressors and illnesses typically induce a reduction in expression of ‘low-resilience behaviors’ such as grooming [7,8], which are not deemed essential for survival (despite being important for the animals). Reductions in these types of behaviors have been used to identify dairy cows that are at increased risk for social stress or disease and thus compromised welfare. For instance, accumulated evidence suggests that cows reduce their use of a mechanical brush, a resource that they are highly motivated to access [9], when experiencing known stressful events [10,11] and negative health events [8,12,13]. Overall, these results are consistent with the idea that brushing is rewarding and that the expression of self-grooming behaviors (using a mechanical brush) generally drops in response to stressors.

The aim of this study was to explore whether the myriad of post-partum stressors experienced by dairy cows after parturition induced a decline in the use of a mechanical brush. In Experiment 1, we first explored whether cows reduced their use of a mechanical brush after parturition (compared to prepartum values) when subjected to the stressors typically experienced by cows at this time (including calving, separation from the calf, regrouping, and introduction to the milking routine). We used younger cows giving birth for the first time (i.e., primiparous) as they are believed to be more vulnerable to stressors around calving [14,15]. We predicted that these cows would increase their latency to use the brush and decrease their use of the brush post-partum compared to the week before calving. We also expected that cows would return to their baseline (pre-partum) levels of brush use in the weeks following parturition. 

In Experiment 2, we specifically explored whether separation from the calf would result in reduced use of the mechanical brush by comparing animals that were subjected to all stressors similarly, except for cow–calf contact and separation. This practice is likely to be a primary stressor for post-partum cows (for review, see [3]). Common practice on most dairy farms is to permanently remove the new-born calf within 24 h after birth, although some organic systems allow longer periods of cow–calf contact. There is now growing interest in systems providing part-time contact [16]. In this experiment, cows were either separated from their calf immediately after calving (early permanent separation treatment) or allowed 29 d of contact (part-time contact treatment). Cows in the latter treatment were allowed 24 h of full contact and then separated from their calf every morning starting on the second day post-partum and reunited every afternoon until day 29. We hypothesized that cows in both treatment groups would increase their latency to use the brush and decrease their use of the brush post-partum. However, given that previous work showed that the emotional bond needs time to become established [17,18,19], we expected a greater decline in brush use in cows that were given more time to bond with their calf and were tested after a shorter time since separation compared to early-separated cows (6 h vs. 24 h). We also expected to see a second decrease in brush use in the part-time contact treatment on d 30 when cows were permanently separated from their calf. 

## 2. Materials and Methods

This study was conducted at the University of British Columbia (UBC) Dairy Education and Research Center (Agassiz, BC, Canada). All procedures were carried out in accordance with relevant guidelines and regulations (Canadian code of practice for the care and handling of dairy cattle-DFC-NFACC, 2016) and were approved by the UBC Animal Care Committee (AUP A15-0117). No animals were subjected to an avoidable stressful procedure, although the intensity of separation distress may have been greater in the part-time contact cows. Given the standard farm practices of the UBC Dairy Education and Research Center, we were required to separate all cows from their offspring at the end of the experiment. To minimize the distressful effects of separation, we ensured that all calves were nutritionally independent of the dam (i.e., they knew how to drink from a milk feeder). 

### 2.1. Animals and Housing

In Experiment 1, 30 primiparous Holstein dairy cows (mean ± SD) 2.0 ± 0.1 years old were enrolled 42 d before parturition and followed until 42 d after parturition. In Experiment 2, 24 Holstein dairy cows (4.3 ± 1.9 years old; parity: 3.1 ± 1.6; 20 multiparous and 4 primiparous cows) were enrolled (pseudo-randomly allocated in two treatment groups: part-time contact: *n* = 11, early permanent separation: *n* = 13) 24 d before parturition until 30 d after parturition. Animals had to be 7.5 mo pregnant and healthy at the time they entered either of the two experiments. Animals had *ad libitum* access to food and water and returned to the routine farming schedule once the experiments ended. The animals’ health was checked daily by the farm staff and bi-weekly by the herd veterinarian (unless clinical signs of illness were noticed by the farm staff).

Animals in both experiments were kept in pens (12 m × 8 m) containing 12 sand-bedded lying stalls (120 cm wide × 260 cm long) and 16 feeding spaces. Although the number of animals per pen varied due to unpredictable calving dates, the maximum never exceeded 12. Animals had ad libitum access to water and a diet formulated according to [20] containing 25.1% rye straw, 34% grass hay, 40.5% grass silage, and 0.5% minerals for the first 21 days of the study. Due to the increased nutritional demands in the last stages of pregnancy, the diet was reformulated three weeks before parturition as follows: 27.3% rye straw, 47% corn silage, and 26% mash. Post-partum cows were fed 4.6% alfalfa hay, 11.25% grass hay, 45.3% corn silage, and 30% mash.

### 2.2. Separation from the Calf and Post-Partum Housing

In Experiment 1, when imminent signs of calving were present (i.e., udder enlargement, milk letdown, relaxation of tail ligaments), cows were individually moved to a straw-bedded maternity pen (2 m × 12 m). After parturition, animals were separated from their calf within (mean ± SD) 1.7 ± 1.8 h of birth. Cows were taken to the milking parlor approximately 6 ± 3.7 h after calving. After milking they were moved to a new pen of lactating animals (48 lying stalls and 48 feed-bunk spaces; four pens where the gates between the pens had been removed, and each pen was identical in size to the pen described above).

In Experiment 2, when signs of calving were present (as described above), cows were moved to a maternity pen that consisted of an alley and a sawdust-bedded pack (2 m × 7 m). At calving, animals were separated as described above (early permanent separation treatment) or allowed extended contact including suckling (part-time contact treatment). Cows were kept in the maternity pen for approximately 24 h after calving, regardless of treatment, and then moved to the post-partum pen. Cows in the part-time contact treatment had full contact with the calf for approximately 24 h after birth (except during milking). Cow and calf were then separated during the day and allowed contact during the night, similar to that undertaken in previous studies (e.g., [21]). This experimental design was chosen for three reasons. First, it enabled us to avoid separating cows from their calves just before testing for brush use. Second, it allowed us to house calves and cows in free stalls without increasing the risk of injuries to calves as the automatic manure scrapers were run when the calves were not present. Third, it allowed us to teach calves how to drink from a milk bar, ensuring that they would be nutritionally independent when separation eventually took place. Every day, at 1830 h, when the cows returned from milking, part-time contact cows entered an adjacent pen where they were reunited with their calf; whereas early separated cows returned directly to the pen used for daytime housing. This schedule continued until day 29 when part-time contact cows were permanently separated from their calves.

### 2.3. Brush Test

In both experiments, for the cow to be considered the experimental unit, the brush was located outside of the home pen and cows were tested individually. Cows only had access to the brush during testing. All enrolled cows were first habituated to the process of moving from the home-pen to the alley (15 m × 3 m) where the brush was located. In Experiment 1, all animals were naïve to mechanical brushes. Habituation to the mechanical brush (Lely, Luna, dimensions: 80 cm long × 50 cm wide and placed at 150 cm height) began 6–8 weeks before the expected calving date and consisted of familiarizing two to three cows at a time by moving them from the group to the alleyway with the brush, for 10 min per day for 14 d. Individual testing started in week 5 before parturition and was repeated every 7 d until calving, the last test being done on average (mean ± SD) 6 ± 3.2 d before parturition (day varied due to differences between predicted and actual calving dates). Brush tests (and habituation) were always performed between 1300 and 1500 h. 

After the 14 d habituation period, cows were tested on average (mean ± SD) 5.4 ± 1.2 times before parturition. Cows were then tested again at 2, 7, 14, 21, and 42 d after parturition. We used multi-day intervals between test days to reduce the risk that the cows’ motivation to use the brush was affected by usage during the previous test. We first tested cows 2 d after calving to explore the acute effects of calving and associated stressors and then performed regular testing to explore recovery. Latency to use the brush was scored live at the time of testing. Duration of brushing was recorded using a camera (Panasonic WVCP-470, Newark, NJ, USA) positioned above the brush in Experiment 1 and was scored live in Experiment 2. Video analysis was performed with Geovision viewlog software (Vision systems, Montreal, QC, Canada). In the case of lactating cows, testing always occurred at least 2 h before milking. 

In Experiment 2, habituation to the brush was undertaken as described for Experiment 1, and cows were then tested individually every 6 d beginning 24 d before calving (at least four times) and on days 2, 6, 12, 18, 24, and 30 after calving. Latency to access the mechanical brush and duration of brushing was measured during each test. We elected to keep time since calving constant (to avoid confounding time since calving and testing times), although this means that the response to the first test of separation is confounded by time since separation (i.e., time since separation was about 24 h for the early separated cows and approximately 6 h for cows of the part-time contact treatment).

### 2.4. Statistical Analysis

The individual cow was considered the statistical unit. Power analyses were run using the function ‘pwr’ in R using similar estimates of power and effect size for both outcome measures. Other analyses were done in SAS unless specified. Statistical analyses followed *a priori* predictions and significant interactions were explored by stratification. Analyses were based on animals that were healthy and with no obvious signs of lameness. Clinical lameness assessments were part of our routine health checks, but cows were not routinely gait scored for this study. Statistical codes and dataset are provided in the Supplementary materials 2 and 3, respectively.

#### 2.4.1. Experiment 1

**Figure 1:**
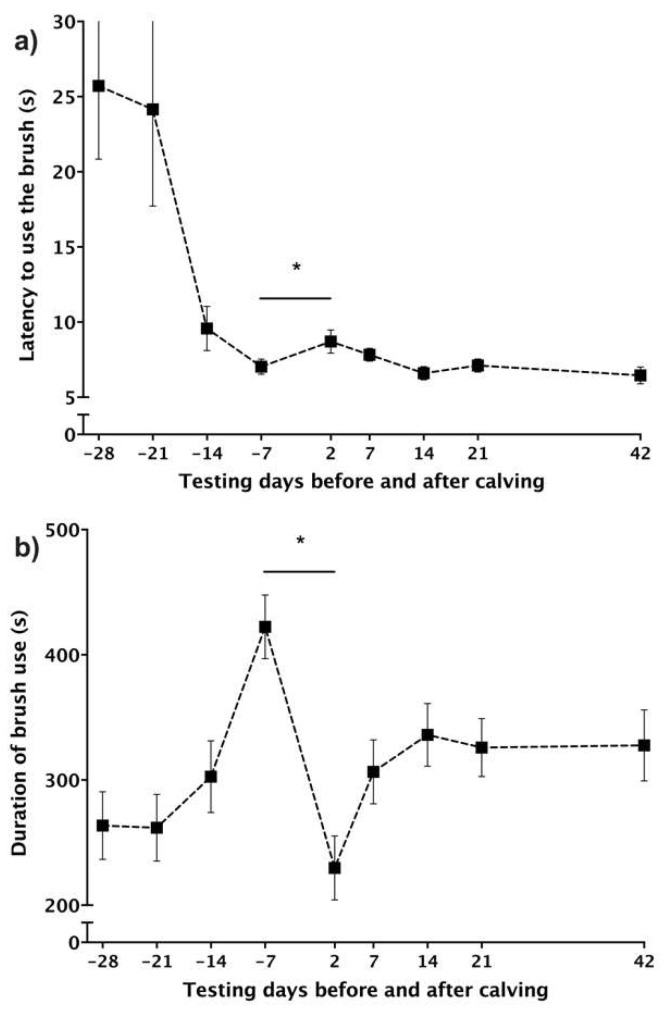
Parturition induces changes in brush use. (**a**) Latency to use the brush (mean ± SE) on days before and after calving (*n* = 26). (**b**) Duration of brush use (mean ± SE) on days before and after calving in primiparous cows (*n* = 26). Cows were progressively habituated to the testing routine potentially explaining the progressive decrease in latency and increase in brush use over the prepartum period. The baseline measures were obtained during the last brush test before calving. Data presented came from Experiment 1. An asterisk (*) represent a significant difference between day-7 and day 2.

A sample size of 24 cows was determined by our *a priori* power analysis (with power set at 0.8, significance at 0.05, and Cohen’s d at 0.6). These were determined to detect a medium effect size; thus, we enrolled 30 cows. One animal became ill, and three animals failed to use the brush during prepartum testing and were therefore excluded from analyses, resulting in a final sample of 26 cows. We used mixed linear models, with either latencies to use the brush or brush use duration as the outcome variable, to test the effect of day relative to calving with cow identity specified as a random effect. The last test before calving was used as the baseline because brush use increased over time pre-partum (Figure 1). Animals seemed to need multiple testing sessions to become used to the testing routine and to learn to use the brush in a consistent way. Normality of the residuals was verified graphically. All cows used the brush within the 600-s test period included in the analysis; latencies were log transformed to improve the normality of residuals. Cohen’s d was used to assess the effect size. We compared post-partum values to baseline values, with the Bonferroni–Holm correction applied in cases of multiple comparisons. Observers could not be blinded to treatment as cows were housed in different pens before and after calving. Inter-observer reliability scores were obtained for the duration of brush use (intra-class correlation coefficient) using a subset of 16 videos scored by an observer who was blind to the study objectives and to treatment. Results showed very good reliability (ICC = 0.98, Cl_95_ = 0.95–0.99).

#### 2.4.2. Experiment 2

Power analyses were again run using the function ‘pwr’ in R. Sample size of 24 individuals was recommended for a power set at 0.8, significance level set at 0.05, and a Cohen’s d equal to 0.6 (medium effect-size). One animal was removed from the analyses due to health issues, resulting in a final sample size of 23 cows. The effect of separation was tested with separate models for the two outcome variables (latency to use the brush and the duration of brush use), testing the effect of day, and the interaction between day and treatment (i.e., early permanent separation and part-time contact), with cow identity included as a random effect to adjust for repeated measures. On the second day post-partum, we expected that cows from both groups would increase their latency to use the brush and decrease the time spent using the brush, with stronger effects in cows provided part-time contact (given that they had more time to bond with their calf and were separated more recently than the early-separation cows). Comparisons were made using the last test prepartum (following the same logic as for Experiment 1). On the 30th day (the day following permanent separation), we also compared latency and brush use with respect to the previous week (day 24), looking for a treatment ∗ day interaction (with cow identity included as a random effect to adjust for repeated measures). We also conducted a second analysis, splitting by treatment and using paired-sample Fisher–Pitman permutation tests. Cohen’s d is reported as a measure of effect size.

## 3. Results

### 3.1. Experiment 1

Consistent with our predictions, cows increased their latency to use the brush postpartum (F_5,122_ = 4.53, *p* < 0.001) compared to the week before parturition; cows were slower to start brushing on day 2 (by 39 ± 15.7% (1.7 ± 0.77 s), t_122_ = 3.06, CL_95_: 0.15 to 3.20, *p* = 0.0027, d = 0.69; Figure 1a). Cows also reduced the time spent brushing postpartum (F_5,124_ = 11.97, *p* < 0.0001); cows used the brush less on day 2 (by 47 ± 5.5% (−199.9 ± 26.3 s), t_124_ = −7.60, CL_95_: (−)252.0–(−)147.9, *p* < 0.0001, d = 1.55; Figure 1b). The time spent brushing after calving did not fully recover (Figure 1b); values stayed below the baseline for all post-partum test days. 

### 3.2. Experiment 2

**Figure 2:**
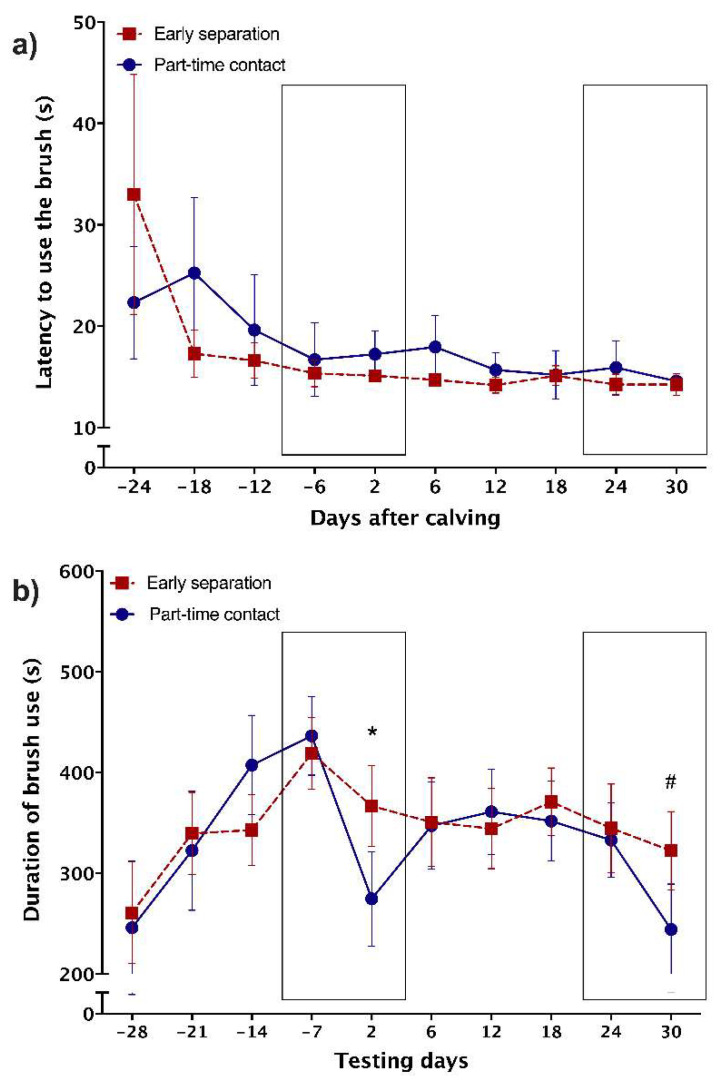
Separation from the calf induced changes in brush use in dairy cows. (**a**) Latency (mean ± SE) to use the brush and (**b**) brush use (mean ± SE) for early permanently separated cows (i.e., permanently separated from their offspring within 2 h of birth; *n* = 12) and part-time contact cows (i.e., allowed 24 h of continuous contact after birth and then 12 h/d of contact for 29 days before permanent separation; *n* = 11). Cows were progressively habituated to the testing routine explaining the progressive decrease in latency and increase in brush use over the prepartum period. Please note that one cow was an extreme outlier for latency to use the brush on days −24, −18, and −12 and was thus not represented in this figure. The last measure preceding calving was used for comparisons regarding the first separation (day 2 postpartum). Similarly, we used data from day 24 as a baseline when testing the effect of separation on day 29 (and tested on day 30). Boxes represent the periods for which statistical comparisons were made to assess the effects of separation. Data presented came from Experiment 2. The asterisk represents a significant day × treatment interaction and the # represents a significant effect of time in the part-time contact treatment.

No differences were found in the latency to use the brush between the week before calving and day 2 post-partum (*p* > 0.05; Figure 2a). Similarly, we found no effect of treatment (F_1,21_ = 0.10, *p* = 0.76) and no interaction between treatment and test day on the latency to use the brush (F_1,19_ = 0.68, *p* = 0.42). However, consistent with the results from Experiment 1, cows from both treatments decreased their use of the brush on day 2 (Estimate = (−)104.5, SE = 17.5, CL_95_: (−)141.1–(−)68.0, F_1,20_ = 35.63, *p* < 0.0001, d = 3.70). Moreover, cows in the part-time contact treatment showed a more pronounced decline in brush use (part-time contact: −40.52 ± 8.51% (−156.80 ± 25.81 s) vs. early permanent separation: −11.35 ± 7.77% (−52.24 ± 23.67 s); day ∗ treatment interaction: F_1,20_ = 8.91, *p* = 0.007, d = 0.90; Figure 2b).

The part-time contact cows were then reunited with their calf following the afternoon milking and spent the night with their calf before being again temporarily separated the following morning. This pattern of contact at night and separation during the day was repeated for 29 d. Cows of the early-separation group were provided with no further contact with their calf. All cows were subjected to the brush test on postpartum days 6, 12, 18, and 24, with no treatment differences detected (all *Ps* > 0.05; Figure 2a,b). On day 29, part-time contact cows were permanently separated (i.e., not reunited with their calf) and all cows were tested the following afternoon (day 30) in comparison with the previous testing day (i.e., day 24). We found no evidence of a time ∗ treatment interaction for either latency to use the brush or duration of brush use (all *Ps* > 0.05). However, graphical analysis revealed that any effect was obscured by between cow variation in the early-separation treatment (see Supplementary Figure S1), so we explored changes in brush use separately for the two treatments. For the part-time contact cows, we found a decline in brush use on day 30 relative to day 24 (by 29.40 ± 12.40%; Z = 2.38, n = 11, *p* = 0.008, d = 1.31), but no changes were detected between day 24 and day 30 for cows in the early separation treatment (*p* > 0.05). 

## 4. Discussion

The aim of this study was to assess whether cows would reduce their use of a mechanical brush after experiencing parturition and separation from their calf compared to brush use in the week before calving. Cows showed a reduced use of a mechanical brush in the days after parturition and did not return to the prepartum level of brush use after calving. In addition, cows showed a drop in brush use after separation from their calf on day 2 and day 30 postpartum. This reduction in low-resilience behaviors indicate that the postpartum period and separation from the calf challenge the welfare of dairy cows. 

The decline in motivation and use of the mechanical brush after parturition and separation from the calf are consistent with previous results indicating that dairy cattle reduce their use of a mechanical brush when experiencing negative affective states [10] and health issues [8,12,13]. However, contrasting results exist. Pregnancy examination and bovine respiratory disease did not lead to reduced use of a freely available mechanical brush [11,22]. These differences may be explained by the nature of the negative experience or by other factors such as the location of the brush [11].

Although one previous study showed an increase in brush use during the post-partum period [23], cows were not provided with brush access before calving, preventing any test of the effect of calving itself. Here, cows experiencing parturition for the first time were likely affected by the diverse stressors (e.g., parturition and associated pain, separation from the calf, introduction to the milking routine, and social mixing with unfamiliar conspecifics) associated with this period. Experiment 1 did not aim at disentangling which specific stressors were affecting cows. In contrast with previous results showing a reduction in brush use only on the day of social mixing [10], the current study also found that cows changed their brush use for weeks following parturition. This suggests that post-partum stressors may in fact induce more severe welfare issues than those observed after social mixing alone. However, it is interesting to note that not all cows expressed a similar pattern of brush use postpartum, with some recovering faster than others. Future studies should explore what factors contribute to increased vulnerability postpartum; ease of calving [24] or personality traits [25] are two factors worthy of more investigation.

In Experiment 2, most cows reduced their use of the brush after calving (when tested on day 2) but, unlike in Experiment 1, we did not observe an increase in latency to use the brush. Although comparisons are difficult because of differences in methodology between the two experiments, we speculate that these differences may be due to the use of primiparous cows in Experiment 1 and mostly multiparous animals in Experiment 2; primiparous cows may have been more sensitive to postpartum stressors given that they have no previous experience with these challenges [15,26]. The differences between the two experiments may also be explained by primiparous cows being at increased risk of difficult calving [24], but we were unable to test this. Although latencies are commonly used to draw inferences about motivation in behavioral studies (e.g., [27]), they may not be ideal to assess motivational deficits in the current context given the limited increase (a few seconds) in Experiment 1 and the absence of effect in Experiment 2. Previous work has used a weighted push gate to assess cow motivation [28]; a similar approach could be used in the future.

Results from Experiment 2 indicate that separation from the calf triggers a reduction in performance of low-resilience behaviors in the dam. Part-time contact cows (who had full contact for 24 h and were separated approximately 6 h before testing) showed a stronger response compared to those that were separated within 2 h of calving and tested on the following day. Our results can be interpreted in two ways: (1) separation from the calf was more distressful for part-time contact cows because they had more time to bond with their calf or (2), they reacted more strongly because separation occurred more recently (approx. 6 h vs. 24 h). Previous work has shown that cows are more responsive to separation when this happens after spending more time with their calf [17,18,19], but we cannot rule out the second interpretation in the current study. Future studies should aim at controlling this confounding factor by using additional treatment groups. This was unfortunately impossible due to time and space constraints. In addition, during the first 24 h of calf contact, cows of the part-time contact group were briefly separated (less than 45 min) from their calf for milking. This additional separation event may have had additional effects on the cows and may have contributed to the differences observed between treatment groups. 

The rebound observed in brush use after day 2 suggests that cows habituated, at least in part, to the partial-contact procedure. However, and consistent with the results of Experiment 1, the cows did not return to prepartum levels of brush use. We chose this design (part-time contact) to avoid separating the cows from their calf just before testing. That said, future studies should explore whether full calf contact provides additional benefits to cows. Moreover, cows that were able to have contact with their calf during the nighttime hours showed a second decline in brush use after permanent separation, when tested on day 30 after calving. Although no effect was found in the early-separation group, some cows in this group also declined (explaining why we did not detect a day ∗ treatment interaction). We did not expect such variability a month after calving and were probably under-powered to detect the interaction. For this reason, we decided to split the data by treatment group for our secondary analysis.

One limitation of our study is that brush use was not consistent prepartum; the progressive decrease in latency and increase in brush use over the four-week period suggests that cows were still learning to use the brush and getting used to the testing routine. This result is consistent with previous reports, indicating that cows need time to master the use of the brush [22], and need many pre-testing sessions to obtain consistent measures when the brush is provided for limited periods [10]. One challenge in the current study is that cows were tested alone, and this may increase the time required to learn to use the brush effectively (most animals used it on the head and the neck before discovering it could be used on the back). It is also possible that brush use increased prepartum for other reasons. Future studies should give more time to cows to use the brush (or additional training sessions) to obtain consistent measures before assessing changes, allowing the use of multiple days for a stable baseline estimate.

Considering that the cows’ physical (e.g., diet, pens, milking routine) and social environment (e.g., social mixing) change immensely between the prepartum and postpartum stages, it is difficult to draw inferences on the cow’s experience based upon home-pen behavioral changes postpartum (e.g., feeding behaviors). Changes in brush use may be the result of a reduction in general activity due to negative energy balance, although we suggest that this explanation is unlikely. Previous studies have reported that primiparous cows increase their activity after calving (e.g., [29]). In addition, we tested animals individually and in a specific and familiar area, away from the home-pen and from other sources of disturbance. Cows had access to the brush in this environment for only 10 min and did not have to compete for access. In these conditions, using the brush was not costly. A change in time budget or energy state were unlikely to be responsible for the drop in brush use observed in both experiments, especially 30 days after calving.

The persistent drop in the use of the brush after parturition was likely due to the cumulative effects of postpartum stressors, similar to what was observed in studies designed to induce depression in laboratory animals [30]; depressive-like states have been noted in other species such as rats when subjected to pain [31], social stress [5], and separation from offspring [6]. Cows are subjected to a myriad of stressors at parturition, any or all of these factors may have been important. Given that cows in Experiment 2 were subjected to the same post-partum changes with the exception that half were not immediately separated from their calf, our results suggest that the observed drop in brush use was most likely stress induced. This result is consistent with previous studies showing that separation is stressful [32,33] and that cows respond more strongly to separation when given time to bond with their calf [17,18,19].

The drop in use of the brush may reflect an anhedonia-like state. Anhedonia, defined as “deficits in the hedonic response to rewards (“consummatory anhedonia”) and a diminished motivation to pursue them (“motivational anhedonia”)” [34], is associated with the experience of acute or chronic stressors [35] and is one of the most studied behavioral changes associated with negative mood in animal models [36,37]. Whether captive animals experience depressive-like states is attracting more attention [38] and recent work provides some evidence of anhedonia-like responses in farm species. For instance, pain due to hot-iron disbudding in dairy calves was associated with reduced time spent playing [39], decreased motivation for milk [40], and reduced consumption of a sweet solution [41]. 

Taken together, these results suggest that the use of rewarding resources (e.g., sweet solutions) or activities (e.g., brushing) can be used to detect the effects of routine farm procedures on affective states [42]. We call for more animal welfare research on cow-calf contact systems given cows motivation for providing maternal care [43].

## 5. Conclusions

Dairy cows reduced their use of a mechanical brush postpartum. Separation from the calf also induced reduction in the use of the brush. These results are consistent with the idea that these stressors induce negative affective states in dairy cows. 

## Data Availability

Data and codes are freely available and included in the Supplementary Materials.

## Supplementary Materials

The following are available online at https://www.mdpi.com/article/10.3390/ani11113031/s1, Figure S1: Separation from the calf induces changes in brush use. Supplementary Material 2: Statistical codes, Supplementary Material 3: Dataset.
