# Supplementary material for: Postpartum Stressors Cause a Reduction in Mechanical Brush Use in Dairy Cows
Source: Animals (Basel). 2021 Oct 22;11(11):3031. doi: 10.3390/ani11113031 (PMC8614528; doi:10.3390/ani11113031)
Supplement: Supplementary file 1 [file animals-11-03031-s001.zip › Supplementary Material 1.pdf]

Postpartum stressors cause a reduction in mechanical brush use in dairy cows

Benjamin Lecorps, Allison Welk, Daniel M. Weary, Marina A.G. von Keyserlingk*

Animal Welfare Program, Faculty of Land and Food Systems, 2357 Main Mall, University of British Columbia, Vancouver BC V6T 1Z6

*Corresponding author email: marina.vonkeyserlingk@ubc.ca

Supplementary material 1:

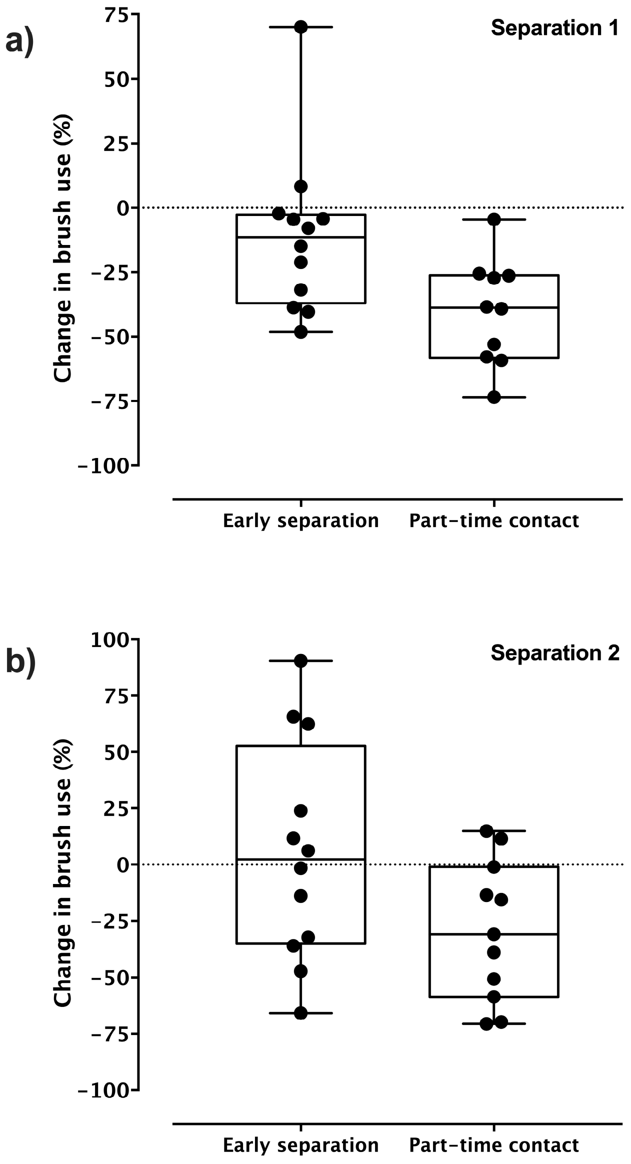

Supplementary Figure S1. Separation from the calf induces changes in brush use. Change in brush use on separation occurring on day 2 (a) and on day 30 (b) postpartum. Data presented come from Experiment 2.
