# Supplementary material for: Postpartum Stressors Cause a Reduction in Mechanical Brush Use in Dairy Cows
Source: Animals (Basel). 2021 Oct 22;11(11):3031. doi: 10.3390/ani11113031 (PMC8614528; doi:10.3390/ani11113031)
Supplement: Supplementary file 1 [file animals-11-03031-s001.zip › Supplementary Material 2.pdf]

Postpartum stressors cause a reduction in mechanical brush use in dairy cows

Benjamin Lecorps, Allison Welk, Daniel M. Weary, Marina A.G. von Keyserlingk*

Animal Welfare Program, Faculty of Land and Food Systems, 2357 Main Mall, University of British Columbia, Vancouver BC V6T 1Z6

*Corresponding author email: marina.vonkeyserlingk@ubc.ca

Supplementary material 2:

Statistical codes

To run in SAS

```
FILENAME REFFILE ‘File directory’;

PROC IMPORT DATAFILE=REFFILE
DBMS=XLSX
OUT=Experiment1;
GETNAMES=YES;
RUN;

data numbers;
set Experiment1;
run;

PROC CONTENTS;
RUN;

data Baseline_D2;
set Experiment1;
if day in ('6','12','18','24','30','36') then delete;
run;


*Produce descriptive statistics LOG Lat;
proc means data=Baseline_D2 nmiss mean std stderr lclm uclm median min max qrange
maxdec=2;
var Day0_log_lat Day2_log_lat;
run;

*Test for the normality of the differences;
```

```
proc univariate data=Baseline_D2 normal;
var Diff_log_lat;
run;

*Run the Paired T-test;
proc ttest data=Baseline_D2 plots=all;
paired Day2_log_lat*Day0_log_lat;
run;


*Produce descriptive statistics Brush use;
proc means data=Baseline_D2 nmiss mean std stderr lclm uclm median min max qrange
maxdec=2;
var Day0_brush Day2_brush;
run;

*Test for the normality of the differences;
proc univariate data=Baseline_D2 normal;
var Diff_brush;
run;

*Run the Paired T-test;
proc ttest data=Baseline_D2 plots=all;
paired Day2_brush*Day0_brush;
run;
```

Effects of first separation

```
FILENAME REFFILE ‘File directory’;

PROC IMPORT DATAFILE=REFFILE
DBMS=XLSX
OUT=Experiment2;
GETNAMES=YES;
RUN;

data numbers;
set Experiment2;
run;

PROC CONTENTS;
RUN;

proc sort;
```

```
by Day;
run;
proc univariate plot normal;
var Lat_brush Log_lat_brush Brush ;
run;

data Separation1;
set Experiment2;
if day in ('6','12','18','24','30','36') then delete;
run;


** SEPARATION 1 **
*Log_latencies ;
Proc mixed data = Separation1;
Class Animal day Treatment;
Model Log_lat_brush = day Treatment day*Treatment/ residual  ;
Repeated day / subject =Animal type=ar(1);
Lsmeans day/cl diff;
Run;

*Brush use;
Proc mixed data = Separation1;
Class Animal day Treatment ;
Model Brush = day Treatment day*Treatment / residual  ;
Repeated day / subject =Animal type=ar(1);
Lsmeans day Treatment / cl diff;
Run;
```

Effects of permanent separation
To run in R

```
# Libraries to install
library(lmPerm)
library(ICC)
library(FactoMineR)
library(coin)
library(car)
library(lme4)
library(pgirmess)
library(outliers)
library(emmeans)
library(pwr)

########################################
# Power calculation
```

```
#pwr.2p.test	two proportions (equal n)
#pwr.2p2n.test		two proportions (unequal n)
#pwr.anova.test		balanced one way ANOVA
#pwr.chisq.test		chi-square test
#pwr.f2.test	general linear model
#pwr.p.test	proportion (one sample)
#pwr.r.test	correlation
#pwr.t.test	t-tests (one sample, 2 sample, paired)
#pwr.t2n.test	t-test (two samples with unequal n)

# Experiment 1
pwr.t.test(n=NULL, d=0.6, power=0.8, sig.level=0.05, type=c("paired"))
#n = 24

# Experiment 2
pwr.anova.test(k = 2, n = NULL, f = 0.4, sig.level = 0.05, power = 0.8)
#n = 25

###################
Experiment2<-read.csv(file.choose(), sep=";", dec=",") # File opening
str(Experiment2) #

# Effects of second separation using non-parametric tests

# Create a subset including only Day 24 (baseline) and Day 30 (post-separation)
Exp2_24_30<-subset(Experiment2, Experiment2$Session=="24" |
Experiment2$Session=="30")

# Create a subset including only late-separation cows
Exp2_24_30Late<-subset(Exp2_24_30,Exp2_24_30$Treatment=="late-separation")

# Create a subset including only early-separation cows
Exp2_24_30Early<-subset(Exp2_24_30,Exp2_24_30$Treatment=="early-separation")

# Run Fisher-Pitman permutation tests
# Late separation cows
oneway_test(Lat_brush~factor(Session)|factor(Animal),data=Exp2_24_30Late,distributio
n=approximate (B=100000))
plot(Lat_brush~factor(Session),data= Exp2_24_30Late)
# ns
oneway_test(Brush~factor(Session)|factor(Animal),data=Exp2_24_30Late,distribution=a
pproximate (B=100000))
plot(Brush~factor(Session),data= Exp2_24_30Late)
# Z = 2.3864, p-value = 0.008 *
```

```
# Early separation cows
oneway_test(Lat_brush~factor(Session)|factor(Animal),data=Exp2_24_30Early,distribution=approximate (B=100000))
plot(Brush~factor(Session),data=Exp2_24_30Early)
# ns
oneway_test(Brush~factor(Session)|factor(Animal),data=Exp2_24_30Early,distribution=approximate (B=100000))
plot(Brush~factor(Session),data=Exp2_24_30Early)
# ns
```
